# Video‐Assisted Intralesional Curettage for Invasive Nonmalignant Giant Cell Tumors

**DOI:** 10.1002/atn2.70300

**Published:** 2026-10-09

**Authors:** George Loria, Mohamed Ahmed Mohamed, George Alexander Rublev, Irakli Gogua, Giorgi Zimlitski, Mikheil Zimlitski

**Affiliations:** ^1^ Center for Arthrology, Sports Medicine, and Traumatology, MediClubGeorgia Tbilisi Georgia; ^2^ Department of Orthopaedic Surgery AIETI David Tvildiani Medical University Tbilisi Georgia

## Abstract

Giant cell tumor of bone is a locally aggressive benign neoplasm with a strong tendency for local recurrence after surgical treatment. Intralesional excision and curettage is preferred in most cases to maximize tumor removal while preserving the affected joint. The present technical note describes Video‐Assisted Intralesional Curettage of Invasive Nonmalignant Giant cell tumors, a technique in which intracavitary 30° arthroscopic visualization is integrated with extended intralesional curettage. After the creation of a cortical window for Campanacci grade 2 lesions or the use of the existing cortical defect for grade 3 lesions, the arthroscope is introduced and rotated through 360° to identify residual pigmented tumor in recesses not visible to the unaided eye. Targeted recurettage, high‐speed burring, and monopolar electrocautery on spray mode are then applied under direct video visualization, followed by pulsed saline lavage and reconstruction with polymethyl methacrylate cementation, with the aim of reducing residual microscopic disease at every step.

## Graphical abstract

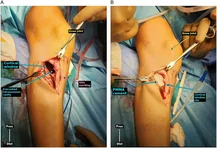

**VIDEO 1:** The video begins with visualization of the tumor cavity by inserting the arthroscope into a previously created cortical window in the anterior proximal tibia. After careful 360° visualization, an electrocautery probe is inserted to ablate parts of the cavity, ensuring comprehensive coverage of any remaining tumor cells. A jet of saline is used throughout the procedure to wash out the bony cavity while dislodging any pathological tissue remnants. A dry visualization of the cavity is performed after irrigation to ensure no cortical breaches or suspicious remnants are present before proceeding with reconstruction using bone cement. Video content can be viewed at https://doi.org/10.1002/atn2.70300.

The unpredictable behavior of giant cell tumor of bone (GCTB), and their tendency to recur, poses a difficult obstacle for treatment in orthopaedic surgery.^1^, ^2^ It is an aggressive benign tumor, representing 4% to 5% of all bone tumors, that frequently involves the metaepiphyseal regions of long bones.^2^ GCTBs display female preponderance and occur after skeletal maturity, with the highest incidence during the third and fourth decades of life.^3^, ^4^, ^5^ Morphologically, the lesion is composed of multinucleated giant cells surrounded by mononuclear stromal cells.^2^ The incidence of malignant transformation among patients with benign GCTB is estimated to be 2% to 11%.^6^

A consensus on the optimal surgical treatment for GCTB is still lacking; however, multiple approaches, including intralesional curettage, wide excision, prosthetic replacement, bone grafting, and adjuvant addition, have been used.^1^ Intralesional excision and curettage is preferred in most cases to maximize tumor removal while preserving the joint, but when employed alone, it has a high recurrence rate of 27% to 82%.^1^, ^3^, ^4^ The recurrence rate after intralesional curettage is lowered to 10% to 20% with concurrent use of power burrs and local adjuvants.^2^ Wide excision has lower recurrence rates (0%‐5%); however, it increases surgical complications and functional impairment.^4^ The objective of this article is to describe a Video‐Assisted Intralesional Curettage of Invasive Nonmalignant Giant cell tumors (VAICING) as an alternative approach to treating GCTB.

## SURGICAL TECHNIQUE

### Preoperative Planning

Patients undergo magnetic resonance imaging and plain radiologic imaging preoperatively. Radiographic signs of a GCTB include the presence of an osteolytic lesion with increased bone remodeling and cortical thinning accompanied by endosteal erosions and indentations.^7^, ^8^ The Campanacci grading system, based on radiological findings, is used for tumor grading and staging.^6^ We use the technique in patients with radiologic findings of cortical erosions with clear nonsclerotic medullary margins (Campanacci grade 2) or patients with complete disruption of the cortex with soft tissue and joint invasion (Campanacci grade 3).^7^, ^9^ Magnetic resonance imaging is the imaging modality of choice because it enables a multidimensional view and superior resolution of tumor margins and cortical changes, often undetected by plain radiographs.^9^ Additionally, magnetic resonance imaging allows for the preoperative planning of the VAICING procedure.^9^ A closed biopsy procedure is performed preoperatively for a definitive diagnosis.^6^ In all patients, immunohistochemistry reveals CD‐68‐positive osteoclastic cells, multinucleated giant cells surrounded by a stroma of p63‐positive mononuclear cell infiltrates, and focal areas expressing cyclin D1.

### Surgical Technique

Table 1 lists all of the equipment used during the VAICING procedure. The procedure begins with optimal retraction of soft tissue followed by complete exposure and visualization of the tumor cavity by creating a cortical window to fully expose and visualize grade 2 tumors^10^, ^11^, ^12^, ^13^ (Figure 1, Video 1). This allows for the best possible access to the major bulk of the tumor.^11^ For grade 3 tumors, a direct approach is undertaken from the site of the existing cortical break created by the growing tumor^11^ (Figure 2). The retracted tissues are covered with hydrogen peroxide‐soaked 4 × 4 mops to avoid tumor cell spillage.^1^, ^10^

**TABLE 1:** Equipment List for VAICING With the Location for Each Respective Manufacturer

**Equipment**	**Brand/Model**	**Manufacturer**
30° arthroscope	HOPKINS II, 4 mm, 30°	KARL STORZ SE & Co. KG, Tuttlingen, Germany
Light source	Cold Light Fountain	KARL STORZ SE & Co. KG, Tuttlingen, Germany
Camera system and monitor	IMAGE1 S Camera System	KARL STORZ SE & Co. KG, Tuttlingen, Germany
High‐speed burr	Midas Rex MR8 Diamond Burr	Medtronic, Minneapolis, MN
Angled curettes	Assorted Angled Bone Curettes	Zimmer Biomet, Warsaw, IN
Electrocautery unit	System 5000 ESU	Conmed, Utica, NY
Pulsed lavage system	Zimmer Pulsavac Plus	Zimmer Biomet, Warsaw, IN
Bone cement (PMMA)	Zimmer Bone Cement	Zimmer Biomet, Warsaw, IN
Retractors	Hohmann and Army‐Navy Retractors	Zimmer Biomet, Warsaw, IN

PMMA, polymethyl methacrylate; VAICING, Video‐Assisted Intralesional Curettage of Invasive Nonmalignant Giant cell tumors.

**FIGURE 1:**
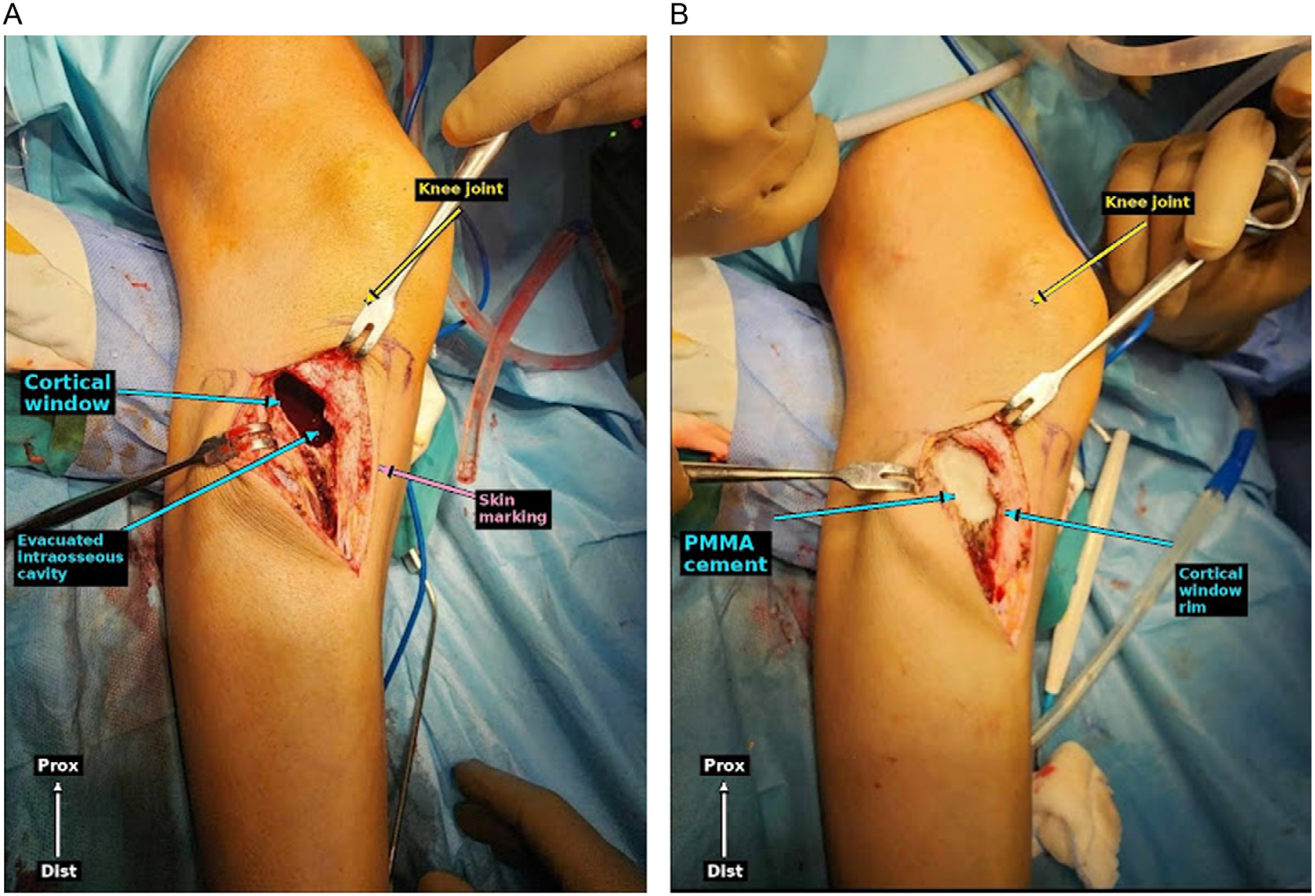
(A) Intraoperative photograph of the right proximal tibia during the “VAICING” technique, with the supine patient's knee flexed to approximately 60° and viewed from the anterolateral aspect (proximal oriented toward the top of the image; orientation marker, lower left). A small ovoid cortical window (cyan arrow) has been created in the anterolateral metaphyseal cortex, lateral to the patellar tendon, exposing the evacuated intraosseous cavity (cyan arrow) after piecemeal curettage, high‐speed burring of the endosteal surface, and adjuvant electrocautery treatment; the knee joint line (yellow arrow) and the planned longitudinal skin incision marking (pink arrow) are shown for orientation. (B) Intraoperative photograph of the same right proximal tibia at the conclusion of the VAICING procedure for GCTB, with the supine patient's knee flexed to approximately 60° and viewed from the anterolateral aspect (proximal toward the top; orientation marker, lower left). PMMA bone cement (cyan arrow) fills the previously curetted intraosseous cavity flush with the cortical window rim, which was placed in the anterolateral cortex lateral to the patellar tendon; the knee joint line (yellow arrow) is labeled proximally to confirm preservation of the tibial plateau and extensor mechanism. (GCTB, giant cell tumor of bone; PMMA, polymethyl methacrylate; VAICING, Video‐Assisted Intralesional Curettage of Invasive Nonmalignant Giant cell tumors.)

**FIGURE 2:**
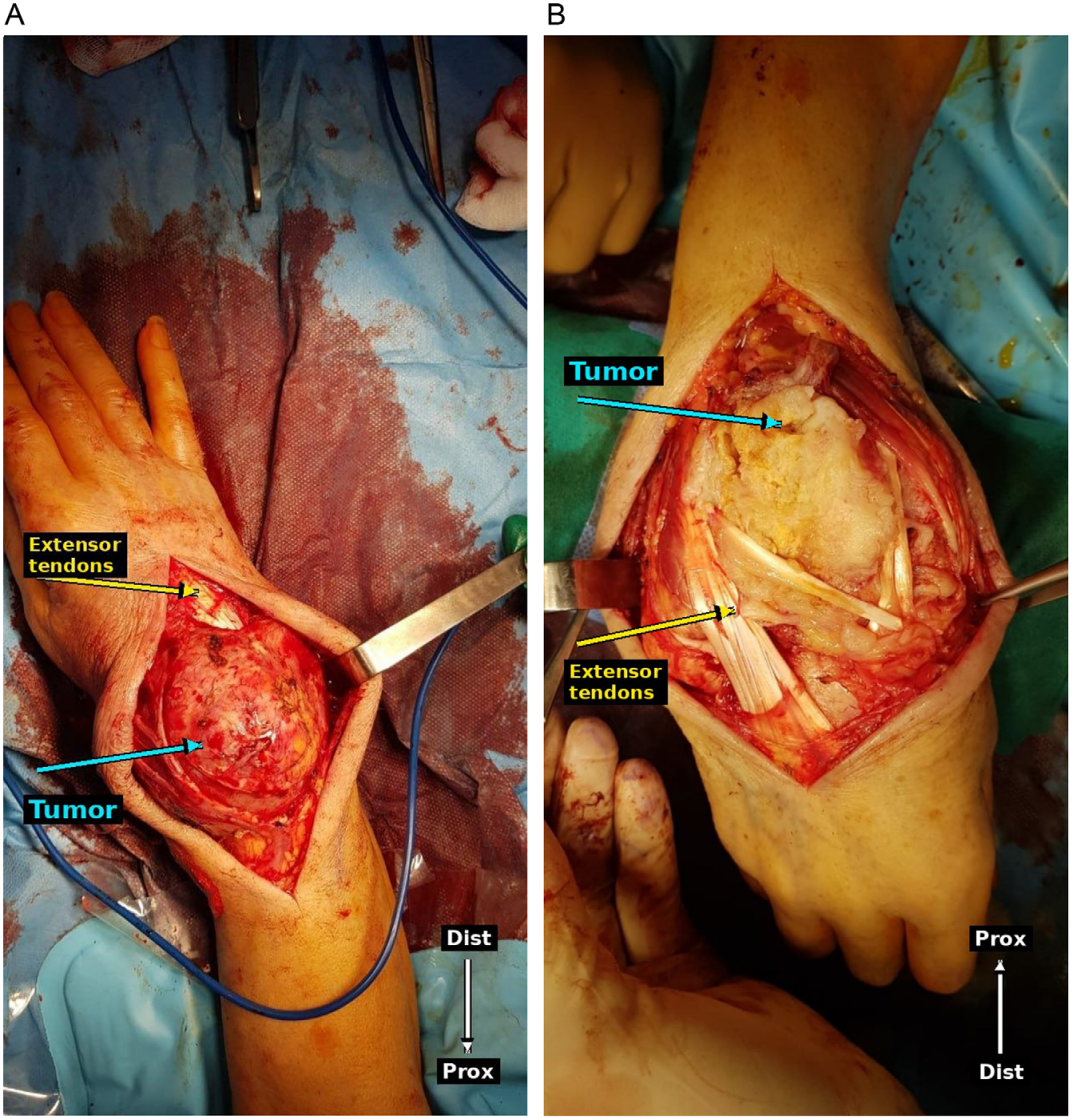
Intraoperative photographs of a Campanacci grade 3 GCTB at the distal radius. The patient is positioned supine with the affected upper extremity on a hand table. A dorsal approach to the distal radius is used. (A) Initial exposure showing the vascular, lobulated GCTB mass protruding through a cortical breach in the dorsal aspect of the distal radius. The extensor tendons are identified and protected with a retractor. The tumor is visible expanding beyond the confines of the native cortex, consistent with Campanacci grade 3 classification featuring complete cortical disruption with soft tissue extension. (B) Deeper exposure after further soft tissue dissection. The yellowish‐white GCTB mass is fully visualized within the expanded distal radius cavity. The extensor tendons are retracted to provide circumferential access for video‐assisted intralesional curettage. The tumor has replaced much of the distal radial metaphysis. In this grade 3 lesion, the surgical approach was directed through the site of the existing cortical break to minimize additional bone destruction while allowing arthroscope‐guided curettage of the cavity. (GCTB, giant cell tumor of bone; PMMA, polymethyl methacrylate.)

A KARL STORZ 30° arthroscope with a light source is introduced through the cortical window and rotated for 360° visualization of the tumor cavity (Figure 3).^14^ Multiple angled curettes of varying sizes are used interchangeably with a high‐speed burr to access as many challenging areas of the lesion as possible while breaking down bony areas within and adjacent to the tumor.^14^, ^15^, ^16^ The high‐speed burr improves the scope of curettage by a few millimeters beyond the grossly visible boundaries of the tumor.^10^ Alternating rounds of electrocautery in spray mode are performed to further minimize recurrence rates (Figure 4).^15^

**FIGURE 3:**
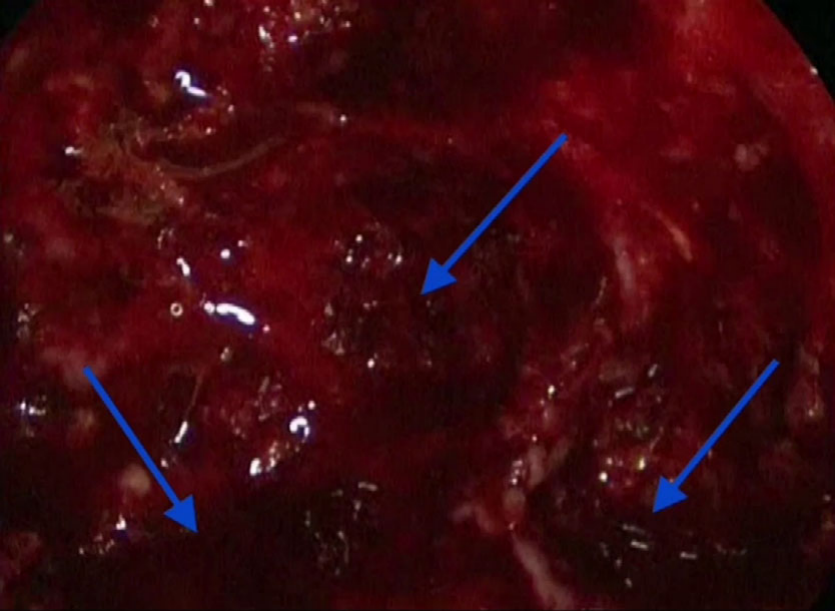
A KARL STORZ 30° arthroscope equipped with a high‐intensity light source is carefully introduced through a premade cortical window. The arthroscope is rotated 360° ensuring complete visualization of the tumor cavity, showing clearly visible lesions composed of giant cell tumor cells (marked by blue arrows).

**FIGURE 4:**
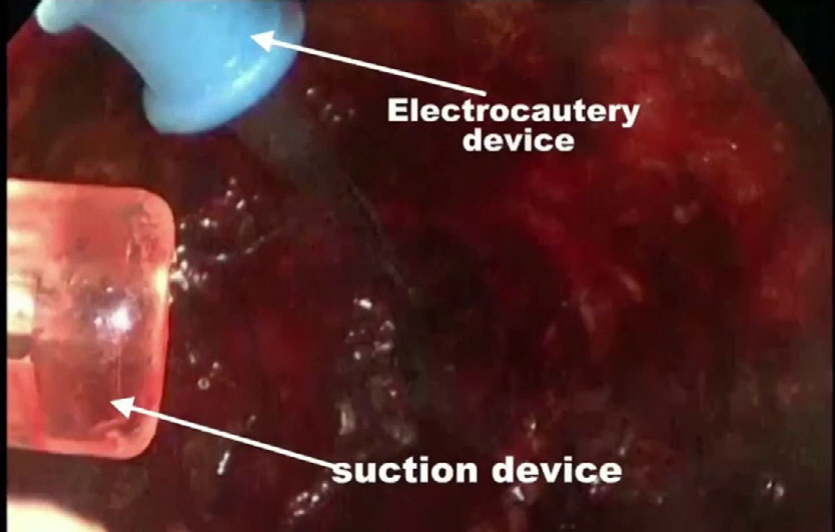
An electrocautery probe (top white arrow) is introduced into the cortical cavity where cauterization is performed under direct arthroscope visualization. The probe is then repositioned to ablate the remaining parts of the cavity, and a suction device is used when necessary to maximize visualization (bottom white arrow).

After alternating rounds of meticulous curettage, electrocautery, and arthroscope insertion, visualization of the normal cortical bone is achieved throughout the bony cavity (Figure 5).^14^ A jet of saline is used throughout the procedure to wash out the bony cavity while dislodging any pathological tissue remnants.^17^ A dry visualization of the cavity is done after irrigation (Figure 6).^14^ Reconstruction of the bony cavity with either bone autografts, bone cement, or a combination of the 2 is performed. Bone allografts are used if the defect requiring repair is <5 mm, and cement is used in instances where the defect is >5 mm.^10^ After achieving hemostasis, layered closure of the incision is performed over a drain.^7^ Range of motion of the affected joints, as tolerated by the patient, is assessed immediately postsurgery.^1^ Patients who undergo surgery for lower limb GCTBs are advised to use crutches for 4 weeks, followed by gradual introduction of weight‐bearing.^1^ Structured physical therapy protocols are implemented based on the surgical site and tailored to each patient via their physical therapist's individual approach.

**FIGURE 5:**
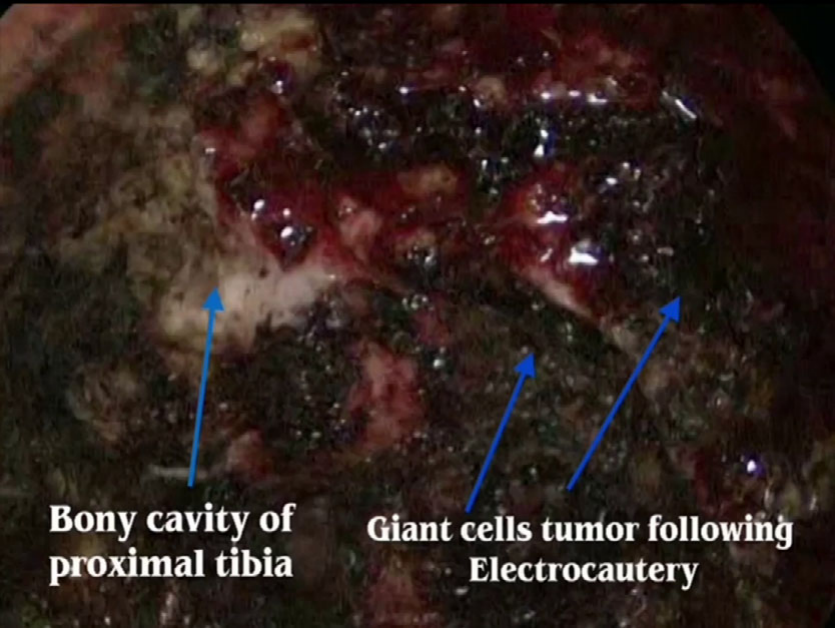
After alternating rounds of cauterization and curettage, the cavity (blue arrows) is visualized again to ensure the presence of normal cortical bone throughout the bony cavity.

**FIGURE 6:**
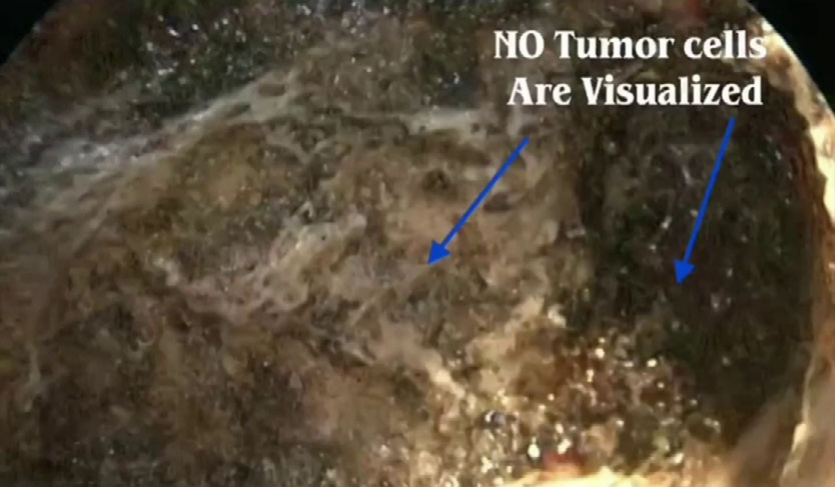
A final dry visualization is performed after irrigation to ensure that the inner surface appears intact, no cortical breaches are observed, and no tumor cells are visualized (blue arrows).

### Postoperative Follow‐Up

Clinical and radiological follow‐up is conducted every 3 months for the initial 2 years and then every 6 months for the next 3 years to check for tumor recurrence and pulmonary metastases.^17^ Functional outcomes and quality of life are assessed using the Musculoskeletal Tumor Society scoring system.^17^ Clinical follow‐up is also used as a monitoring tool for complications such as incision infections, fractures, joint fluid leakage, and joint degeneration.^1^

## DISCUSSION

GCTBs are benign, locally aggressive tumors comprising 4% to 5% of all primary bone tumors.^4^ In around 10% of patients, the tumor may undergo malignant transformation, 2% of which metastasize, primarily to the lungs.^4^, ^5^, ^6^ Patients frequently present with pain which may be accompanied by a restricted range of motion because they frequently arise from the epiphysis which is in close proximity to the joint of the respective bone.^18^ Because of the aggressive nature of GCTB, joint effusion or synovitis might occur caused by joint space invasion.^18^ Twelve percent of GCTB patients present with pathological fractures, which may indicate a more aggressive disease with an increased risk of local recurrence and metastatic spread.^18^ Table 2 highlights a few advantages and disadvantages of VAICING.

**TABLE 2:** Advantages and Disadvantages of VAICING

**Advantages**	**Disadvantages**
Enhanced 360° visualization of entire tumor cavity via arthroscope, allowing detection of residual tumor in areas not visible to the naked eye	Requires arthroscopic equipment and surgeon familiarity with arthroscopic techniques
Joint‐preserving approach that maintains articular surface integrity and range of motion	Learning curve associated with coordinating arthroscopic visualization with intralesional curettage instruments
Improved completeness of curettage in hard‐to‐reach recesses of the tumor cavity compared with standard loupe‐assisted curettage	May not be suitable for Campanacci grade 3 tumors with extensive cortical destruction involving more than one‐third of the circumference
Reduced local recurrence rates compared with conventional intralesional curettage alone (27%‐82%)	Limited to nonmalignant GCTB; not applicable to cases with confirmed malignant transformation
Combination of visual inspection with high‐speed burr, electrocautery, and jet lavage provides multimodal tumor eradication	Arthroscope lens may become obscured by blood, bone debris, or cautery smoke, requiring repeated irrigation
Lower morbidity and faster rehabilitation compared with wide resection	Long‐term data from randomized controlled trials are currently lacking

GCTB, giant cell tumor of bone; VAICING, Video‐Assisted Intralesional Curettage of Invasive Nonmalignant Giant cell tumors.

The Campanacci grading system, a successor of the Enneking classification, was used for tumor staging. It uses radiographic findings to stage bone tumors and is most often used for GCTBs.^18^ Grade 1 lesions are sharply delineated, with a thin border of mature bone without any cortical disruption or minimal thinning of the cortex.^7^, ^18^ Tumors with relatively clear margins and no sclerosis of the medullary margins but displaying signs of cortical disruption or with only minimal thinning and moderate expansion were classified as grade 2.^7^, ^18^ Grade 3 lesions show blurring of tumor margins with clear signs of cortical destruction and invasion of surrounding soft tissues and joint spaces; these lesions typically indicate a rapidly growing, highly aggressive tumor.^5^, ^7^, ^18^ Our procedure was selected based on radiographic findings showing Campanacci grade 2 tumors or Campanacci grade 3 tumors with a cortical break limited to 1 surface and extending to less than one‐third of its circumference.^2^ Patients’ age and proximity of tumor to the joint were other important factors taken into consideration in undertaking the procedure so as to restore the maximum possible range of motion and overall functionality while reducing postoperative complications when compared with a wide resection strategy.^14^, ^18^

For follow‐up, we used the Musculoskeletal Tumor Society questionnaire because it accounts for a wider range of functional and quality of life outcomes when compared with the Short Form Health Survey and the Knee Injury Osteoarthritis Outcome Score. The Musculoskeletal Tumor Society questionnaire consists of 6 sections each scored from 0 to 5 and a total score of 0 to 30 (can be converted to a 0‐100 score), with higher scores indicating better outcomes.

Over the years, an intralesional anatomy‐sparing approach has led to local recurrence rates ranging from 25% to 50%.^19^ This led to the use of several physical and chemical local adjuvants like polymethyl methacrylate, polymethyl methacrylate with phenol, phenol, and others leading to a successful drop of local recurrence rates to 6% to 25% with a mean time to recurrence of 15 to 16 months.^20^

Table 3 highlights some pearls and pitfalls of the VAICING procedure. The procedure described in our study shows great promise especially in the treatment of Campanacci grade 2 tumors because it spares functionality while minimizing local recurrence.

**TABLE 3:** Pearls and Pitfalls of VAICING

**Pearls**	**Pitfalls**
Use preoperative MRI to map the full extent of cortical involvement and plan the cortical window location	Insufficient cortical window size limits instrument maneuverability and arthroscope range of visualization
Create the cortical window large enough to allow simultaneous passage of arthroscope and curettes for real‐time guided curettage	Failure to cover retracted soft tissues with hydrogen peroxide‐soaked mops risks tumor cell spillage and local seeding
Alternate between curettage, high‐speed burring, and electrocautery in systematic rounds to ensure thorough tumor removal	Relying solely on curettage without adjuvant burring and electrocautery increases the risk of residual microscopic disease
Perform both wet (jet lavage) and dry arthroscopic inspections of the cavity to confirm complete curettage before reconstruction	Skipping the dry visualization step after irrigation may leave residual tumor fragments undetected
For grade 3 tumors, approach directly through the existing cortical defect to minimize additional bone destruction	Aggressive curettage near subchondral bone without visualization risks inadvertent articular surface violation and subsequent joint degeneration
Rotate the 30° arthroscope through the full 360° arc to inspect all recesses, particularly the subchondral region	Overpacking cement into cavities adjacent to articular cartilage can cause thermal necrosis of the subchondral plate

MRI, magnetic resonance imaging; VAICING, Video‐Assisted Intralesional Curettage of Invasive Nonmalignant Giant cell tumors.

## DISCLOSURES

The authors (G.L., M.A.M., G.A.R., I.G., G.Z., M.Z.) declare that they have no known competing financial interests or personal relationships that could have appeared to influence the work reported in this article.
